# Enzymatic Stability and Comparative Effects of Kisspeptin-14, Kisspeptin-13 and Kisspeptin-10 on Beta-Cell Function, Glucose Homeostasis and Appetite Regulation

**DOI:** 10.3390/metabo16090686

**Published:** 2026-09-17

**Authors:** Julie A. Spratt, Neil Tanday, Dearbhla M. McGinn, Chidiebere D. Chukwu, Victor A. Gault, Nigel Irwin

**Affiliations:** Centre for Diabetes, School of Biomedical Sciences, Ulster University, Cromore Road, Coleraine BT52 1SA, Northern Ireland, UK; spratt-j7@ulster.ac.uk (J.A.S.); n.tanday@ulster.ac.uk (N.T.); mcginn-d2@ulster.ac.uk (D.M.M.); chukwu-c1@ulster.ac.uk (C.D.C.); va.gault@ulster.ac.uk (V.A.G.)

**Keywords:** appetite, beta-cell, degradation, glucose tolerance, insulin secretion, islet, kisspeptin

## Abstract

**Background/Objective**: Kisspeptin peptides are being increasingly recognised as regulators of metabolism, but whether these actions differ depending on peptide isoform remains unclear. **Methods**: The current study compared the enzymatic stability and biological effects of kisspeptin-14 (KP-14), kisspeptin-13 (KP-13) and kisspeptin-10 (KP-10) on beta-cell health and function, glucose homeostasis and appetite. Peptide stability was assessed in murine plasma and, following confirmation of KISS1R expression in BRIN-BD11 beta-cells, actions on insulin secretion, beta-cell proliferation and apoptosis were investigated, with additional secretory studies in isolated murine islets. Metabolic effects of KP-14, KP-13 and KP-10 were subsequently evaluated in healthy male and female mice. **Results**: KP-14 was resistant to plasma degradation, whereas KP-13 and KP-10 underwent N-terminal proteolysis, with KP-13 degradation generating KP-10 amongst other fragment peptides. The kisspeptin peptides exerted modest direct effects on insulin secretion from BRIN-BD11 cells, with KP-13 being the most efficacious, a bioactivity profile that was confirmed in islets. In addition, KP-13 significantly enhanced beta-cell proliferation and protected against cytokine-induced apoptosis, producing superior beta-cell proliferative effects compared to exenatide. Acute administration of all kisspeptin peptides elevated circulating glucose concentrations, and while KP-14 and KP-10 impaired glucose tolerance, KP-13 did not. Interestingly, KP-14 enhanced glucose-stimulated insulin secretion, with KP-13 and KP-10 being devoid of such actions. All kisspeptin isoforms suppressed food intake, although only KP-13, and especially KP-10 administration, led to an inhibition of feeding. **Conclusions**: Taken together, these data identify KP-13 as possessing the most favourable overall metabolic actions, based on a combination of beneficial actions on beta-cell health and function together with inhibition of feeding and lack of prominent glucose-elevating actions, suggesting possible therapeutic application for type 2 diabetes.

## 1. Introduction

Kisspeptins are generally considered as neuropeptides with a physiological role in puberty onset and overall modulation of the reproductive system, through interaction with the kisspeptin receptor (KISS1R), also known as GPR54 [1]. More recently, kisspeptin peptides have also become recognised as important hormonal regulators of metabolism [2]. It follows that the reproductive system is intrinsically linked to systemic metabolism, where metabolic hormones such as insulin can both directly and indirectly modulate hypothalamic kisspeptin signalling to help control energy availability and fertility [3]. Furthermore, oestrogen plays a role in pancreatic beta-cell survival and insulin sensitivity [4], whilst testosterone has been shown to influence body composition and glucose metabolism across sexes [5]. In harmony with this, the KISS1R is expressed in metabolically active tissues such as liver and adipose tissue as well as pancreatic islets, where kisspeptin signalling has been implicated in the regulation of insulin secretion, glucose homeostasis and energy balance [6].

However, the reported metabolic effects of kisspeptin peptides have been somewhat inconsistent, possibly reflecting differences in peptide fragment utilised as well as more fundamental divergences in experimental model and study design. For example, kisspeptin has been documented to directly potentiate glucose-stimulated insulin secretion [7,8], whereas others report an inhibitory effect of kisspeptin on pancreatic beta-cell function [9,10]. Kisspeptin has also been postulated to play a positive role in beta-cell growth and survival [11], while also activating beta-cell autophagy that subsequently reduces insulin secretion [12]. There are further mixed messages in terms of the impact of kisspeptin on appetite and food intake, with increased, decreased and no impact on energy intake reported [13,14,15].

Whilst there has been some speculation in terms of species-specific effects of kisspeptin [16], one important, but largely overlooked, determinant of kisspeptin bioactivity is the peptide isoform employed. Thus, kisspeptin is generated through the proteolytic processing of the 145-amino-acid kisspeptin precursor, giving rise to kisspeptin-54 (KP-54), kisspeptin-14 (KP-14), kisspeptin-13 (KP-13) and kisspeptin-10 (KP-10) [17]. All peptide isoforms retain the ability to bind and activate KISS1R [18], with KP-14, KP-13, and particularly KP-10 being more extensively studied owing to smaller size and ease of synthesis. However, the amino acid composition of KP-14, KP-13 and KP-10 is likely to yield distinct differences in enzymatic stability and biological activity, but direct comparisons between the kisspeptin peptides in this regard are limited.

In the current study, we therefore hypothesised that the metabolic actions of kisspeptin peptides are determined, at least in part, by amino acid chain length. Initially, enzymatic stability of KP-14, KP-13 and KP-10 was determined in plasma, followed by confirmation of KISS1R expression in pancreatic BRIN-BD11 beta-cells. Subsequent studies in BRIN-BD11 beta-cells and murine islets included assessment of effects on pancreatic beta-cell secretory function and turnover. Finally, the impact of the kisspeptin peptides on circulating glucose, glucose tolerance, glucose-induced insulin secretion and appetite was evaluated in healthy mice. Through assimilation of these complementary in vitro and in vivo endpoints, we identify kisspeptin fragment peptides with the most favourable metabolic profile as well as providing further insight into the structural determinants underlying the metabolic actions of kisspeptin.

## 2. Materials and Methods

### 2.1. Peptides

All test peptides were purchased from Synpeptide Ltd. (Shanghai, China) in excess of 95% purity. As part of standard in-house procedures, the purity and identity of each peptide was additionally verified in-house using high-performance liquid chromatography (HPLC) and matrix-assisted laser desorption ionisation time-of-flight mass spectrometry (MALDI-TOF MS), respectively, as described previously [19].

### 2.2. Plasma Degradation

KP-14, KP-13 and KP-10 (*n* = 3; 50 μg) were incubated at 37 °C on a plate-shaker in 50 mM triethanolamine/HCl (pH 7.8) with fasted murine plasma (10 μL) for either 0 or 2 h, as previously described [19]. Enzymatic reactions were terminated by addition of 50 μL of 10% (*v*/*v*) trifluoroacetic acid/water (TFA). Reaction mixes were then separated by RP-HPLC using a Kinetex 5 μm C8 column (Phenomenex, Macclesfield, UK) with absorbance monitored at 214 nm. Defined HPLC peaks were collected and identified via MALDI-TOF mass spectrometry (PerSeptive Biosystems, Farmingham, MA, USA).

### 2.3. KISS1R Expression in BRIN-BD11 Cells

To determine the expression of KISS1R/GPR54 in BRIN-BD11 cells (*n* = 3), total RNA was extracted using an RNeasy Mini Kit according to the manufacturer’s instructions (Qiagen, Manchester, UK). RNA (1 μg) was converted to cDNA using a SuperScript II Reverse Transcriptase Kit (Invitrogen, Paisley, UK). Quantitative PCR was performed on a LightCycler 480 using QuantiFast SYBR Green Master Mix (Qiagen, UK), rat GPR54 forward (GGGAACTCACTGGTCATCTT) and reverse (GAGACCTGCTGGATGTAGTT) primers, cDNA and RNase-free water. Amplification conditions were set at 95 °C for initial denaturation, followed by 45 cycles of denaturation at 95 °C, primer annealing at 58 °C and extension at 72 °C, followed by a melting curve analysis over the temperature range of 60–90 °C. Data were quantified using the 2^−ΔΔCt^ method, with normalisation to GAPDH expression.

### 2.4. In Vitro Insulin Secretion

Insulin secretory effects of the KP-14, KP-13 and KP-10 were examined using the well-characterised BRIN-BD11 beta-cell line [20]. For experimentation, BRIN-BD11 cells were seeded into 24-well plates (Falcon Ltd., Thermo Fisher Scientific, Dublin, Republic of Ireland) at a density of 150,000 cells per well. Following overnight attachment, the medium was aspirated and cells were pre-incubated in 1.1 mM glucose Krebs-Ringer Buffer (KRB) for 40 min. The pre-incubation buffer was removed and 1 mL of KRB test solution containing either 5.6 mM glucose alone or 5.6 mM glucose, together with KCl (30 mM), 10^−6^ M exenatide as positive control or kisspeptin test peptides (*n* = 8, 10^−12^ to 10^−6^ M), was then added to the cells. Concentrations of kisspeptin peptides were chosen based on their pico- to nano-molar circulating levels [1] alongside the likelihood of elevated local islet concentrations driven by autocrine or paracrine kisspeptin production, with n numbers based on previous validation when assessing peptide hormone insulinotropic actions [19,20,21]. In addition, the impact of kisspeptin peptides on insulin secretion (*n* = 4; 60 min incubation; 10^−8^ M) from murine islets (14-week-old C57BL/6 mice) isolated by standard collagenase digestion was also examined, as described previously [21]. A subsequent acid–ethanol extraction allowed for the presentation of islet insulin secretion data as a percentage of islet insulin content. For all experiments, following the 20 or 60 min incubation period, supernatant was collected and stored at −20 °C until insulin concentration determination using a dextran-coated charcoal insulin radioimmunoassay [22].

### 2.5. Beta-Cell Proliferation and Protection Against Apoptosis

For all studies (*n* = 3), BRIN-BD11 cells were seeded onto sterilised, clear-glass coverslips (16 mm diameter) and placed in 12-well plates (Falcon Ltd.) at a density of 40,000 cells per well and cultured for 18 h. To examine the impact of exenatide positive control (10^−6^ M) and kisspeptin peptides (10^−8^ and 10^−6^ M) on BRIN-BD11 cell proliferation, Ki-67 primary antibody (Ab15580, AbCam, Cambridge, UK) and Alexa Fluor^®^ 594 secondary antibody were employed, as described previously [23]. To examine potential cytoprotective effects of kisspeptin peptides against apoptosis, cellular stress was induced by the incubation of BRIN-BD11 cells with a cytokine cocktail (IL-1β 100 U/mL, IFN-γ 20 U/mL, TNF-α 200 U/mL) over the 18 h test period, and the rate of apoptosis was monitored through TUNEL staining (Fluorescein, Roche Diagnostics, Burgess Hill, UK). As appropriate, media control and the cytokine cocktail mix alone were employed as negative controls. Fluorescent immunocytochemistry was used to assess proliferation and apoptosis (Olympus system microscope, model BX51; Southend-on-Sea, UK) with a DP70 camera adapter system using DAPI (350 nm), TRITC (594 nm) and FITC (488 nm) filters. To quantify positively stained Ki-67 or TUNEL cells, the cell-counter function within ImageJ Software Version 1.54 (National Institutes of Health (NIH), Bethesda, MD, USA) was used and data were then presented as a percentage of the total number of cells investigated, with approximately 200 cells per treatment group analysed for each of the three biological replicates.

### 2.6. Animals

Groups of normal homozygous lean (+/+) male (*n* = 3) and female (*n* = 3) mice (*n* = 6 in total per group), originally derived from the colony maintained at Aston University, UK [24], were used at 15–18 weeks of age. Use of six animals per group for determination of gluco-regulatory and appetite-suppressive effects of peptide hormones has previously been successfully employed in our laboratory [19,21,23,25,26,27]. All animals were age-matched and housed individually in an air-conditioned room (22 ± 2 °C) with a 12 h light and 12 h dark cycle at Ulster University’s Behavioural and Biomedical Research Unit (BBRU). Animals had free access to drinking water and standard laboratory chow (10% fat, 30% protein, 60% carbohydrate; percentage of total energy 12.99 KJ/g (Trouw Nutrition, Cheshire, UK)), as previously described [25]. All animal experiments were performed according to the UK Animals (Scientific Procedures) Act 1986 covered under a UK Home Office Animal project licence (UK Home Office licence certificate PPL2902 approved on 26 April 2021). Experiments were conducted only by UK Home Office personal licence holders, in accordance with local guidelines from the Ulster Animal Welfare and Ethical Review Body (AWERB) committee as well as adherence to the ARRIVE guidelines for reporting experiments involving animals. Of note, isolated islet studies were performed using C57BL/6 mice, while in vivo studies utilised Aston-derived lean (+/+) male and female mice. Use of different genetic strains allows us to examine the reproducibility of kisspeptin peptide actions across multiple experimental models, enhancing the translatability of our findings. In addition, in accordance with ethical guidelines and to minimise animal use during in vivo studies, a crossover study design was used. The same cohort of 6 unique mice (3 male, 3 female) was used across all treatment arms, with a minimum 4-day washout period.

### 2.7. Glucose Homeostasis

Initially, the impact of kisspeptin peptides (25 nmol/kg bw) on circulating blood glucose concentrations following intraperitoneal (i.p.) injection was investigated in overnight-fasted male and female mice (*n* = 6) using saline vehicle (0.9% *w*/*v* NaCl) control. In a separate series, effects of kisspeptin peptides (25 nmol/kg bw) on glucose tolerance were also evaluated in overnight-fasted male and female mice (*n* = 6) following i.p. injection of glucose alone (18 mmol/kg bw) or in combination with exenatide or kisspeptin peptides (each at 25 nmol/kg bw). Peptide doses were based on our previous extensive investigations and positive outcomes in similar experimental systems using various regulatory peptide hormones [19,23,26,27]. For all experiments, blood samples were obtained from conscious mice via the cut tip of the tail vein and blood glucose was immediately measured using a handheld Ascencia Contour blood glucose meter (Bayer Healthcare, Reading, UK). For glucose tolerance experiments, blood samples were taken from the cut tip of the tail vein at the times indicated in the respective figure, and immediately centrifuged using a Beckman microcentrifuge (Beckman Instruments, High Wycombe, UK) for 30 s at 13,000× *g*. The resulting plasma was then aliquoted into fresh Eppendorf tubes and stored at −20 °C prior to glucose and insulin determination by RIA [22].

### 2.8. Food Intake

Overnight-fasted male and female mice (*n* = 6) were administered an i.p. injection of saline vehicle (0.9% NaCl) alone or in combination with test peptides (each at 25 nmol/kg) and cumulative food intake was measured at regular intervals. All mice were provided with a pre-weighed rodent maintenance diet in their food hopper at t = 0 min, which was then re-weighed at each experimental timepoint.

### 2.9. Statistical Analysis

Statistical analyses were performed using GraphPad PRISM (version 8) software with data presented as mean plus or minus standard error of the mean (SEM). Comparative analyses between groups were carried out using a one-way or two-way analysis of variance (ANOVA), as appropriate, with Bonferroni’s post hoc test. The difference between groups was considered significant at *p* < 0.05.

## 3. Results

### 3.1. KP-13 and KP-10 Are Degraded in Murine Plasma, but KP-14 Is Enzyme-Resistant

Following incubation in murine plasma for 2 h, KP-13 and KP-10 were efficiently degraded (Table 1). In terms of KP-13, we were able to identify the presence of the native peptide, as well as KP-(3-13), KP-(4-13) and KP-(5-13) as distinct degradation products, following 2 h incubation in plasma (Table 1). Degradation products consistent with KP-(2-10) and KP-(3-10) were discovered following 2 h incubation of KP-10 in murine plasma, but we did not detect the native peptide (Table 1). Interestingly, in our system KP-14 was not liable to enzymatic degradation over the 2 h incubation period (Table 1). Corresponding HPLC traces for 0 and 2 h degradation experiments are provided within the Supplementary Material (Supplementary Figures S1–S3).

Peptides (*n* = 3; 50 μg) were incubated with 10 μL fasted murine plasma for 0 and 2 h. Reactions were terminated by addition of oftrifluoroacetic acid (10% *v*/*v*). Degradation profiles were analysed using rp-HPLC with 214 nm UV absorbance and HPLC peaks of interest collected for subsequent MALDI-MS analysis and measurement of molecular mass. Amino acid sequences are provided using standard one-letter notation.

### 3.2. KISS1R mRNA Expression Is Evidenced on BRIN-BD11 Beta-Cells

**Figure 1:**
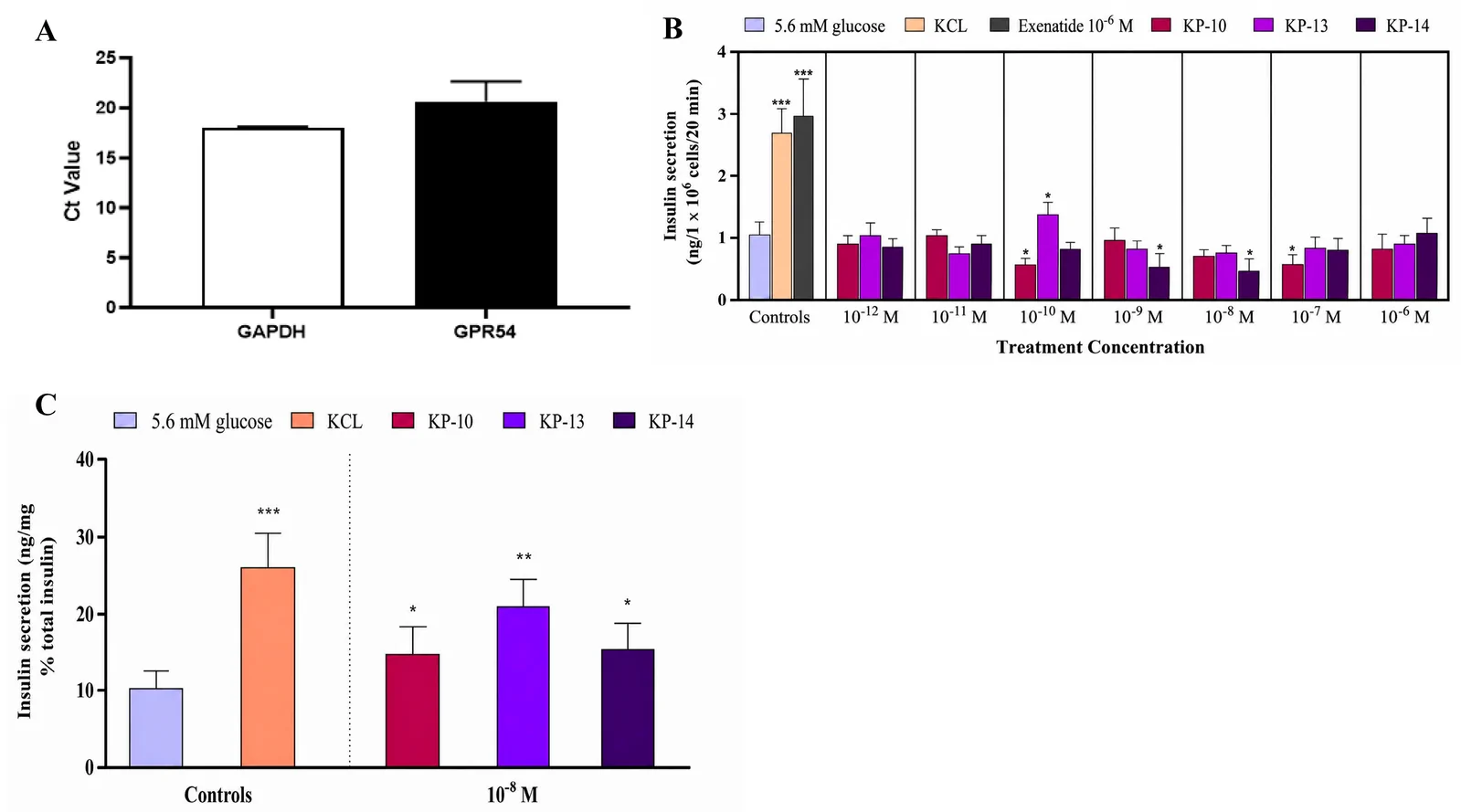
Expression of KISS1R in BRIN-BD11 cells and impact of kisspeptin isoforms on insulin secretion. (**A**) qPCR analysis demonstrating KISS1R mRNA expression in clonal pancreatic BRIN-BD11 cells, normalised to GAPDH. (**B**) BRIN-BD11 cells were incubated (20 min) with 5.6 mM glucose and the effects of KCl (30 mM), exenatide (10^−6^ M) or the kisspeptin peptides (10^−12^ to 10^−6^ M) were evaluated. (**C**) Murine islets were incubated for 60 min with KCl (30 mM) or the test peptides (10^−8^ M) in the presence of 5.6 mM glucose. (**B**,**C**) For all experiments insulin concentrations were measured by RIA. Values are mean ± SEM (*n* = 3 for A, *n* = 8 for B and *n* = 4 for (**C**)). * *p* < 0.05, ** *p* < 0.01, *** *p* < 0.001 compared with respective glucose control.

To confirm the suitability of the pancreatic beta-cell line employed, we initially confirmed detection on KISS1R mRNA in BRIN-BD11 beta-cells by qPCR (Figure 1A).

### 3.3. Kisspeptin Peptide Isoforms Exert Direct Effects on Beta-Cell Insulin Secretion

As expected, the positive controls KCl (30 mM) and exenatide (10^−6^ M) enhanced (*p* < 0.001) insulin release from BRIN-BD11 cells (Figure 1B). Over a concentration range of 10^−12^ to 10^−6^ M, none of the kisspeptin peptides, apart from KP-13 at 10^−10^ M, enhanced insulin secretion (Figure 1B). Indeed, KP-14 and KP-10 were associated with a small inhibitory (*p* < 0.001) effect on insulin secretory function in BRIN-BD11 beta-cells between 10^−10^ and 10^−7^ M (Figure 1B), with KP-14 specifically causing inhibition at 10^−9^ and 10^−8^ M and KP-10 at 10^−10^ and 10^−7^ M. Related observations in isolated murine islets confirmed that 10^−8^ M KP-13 augmented (*p* < 0.01) insulin secretion, having slight, albeit non-significant, improved efficacy over either KP-10 or KP-14 (Figure 1C).

### 3.4. KP-13 Promotes Beta-Cell Proliferation and Partially Protects Against Cytokine-Induced Apoptosis

**Figure 2:**
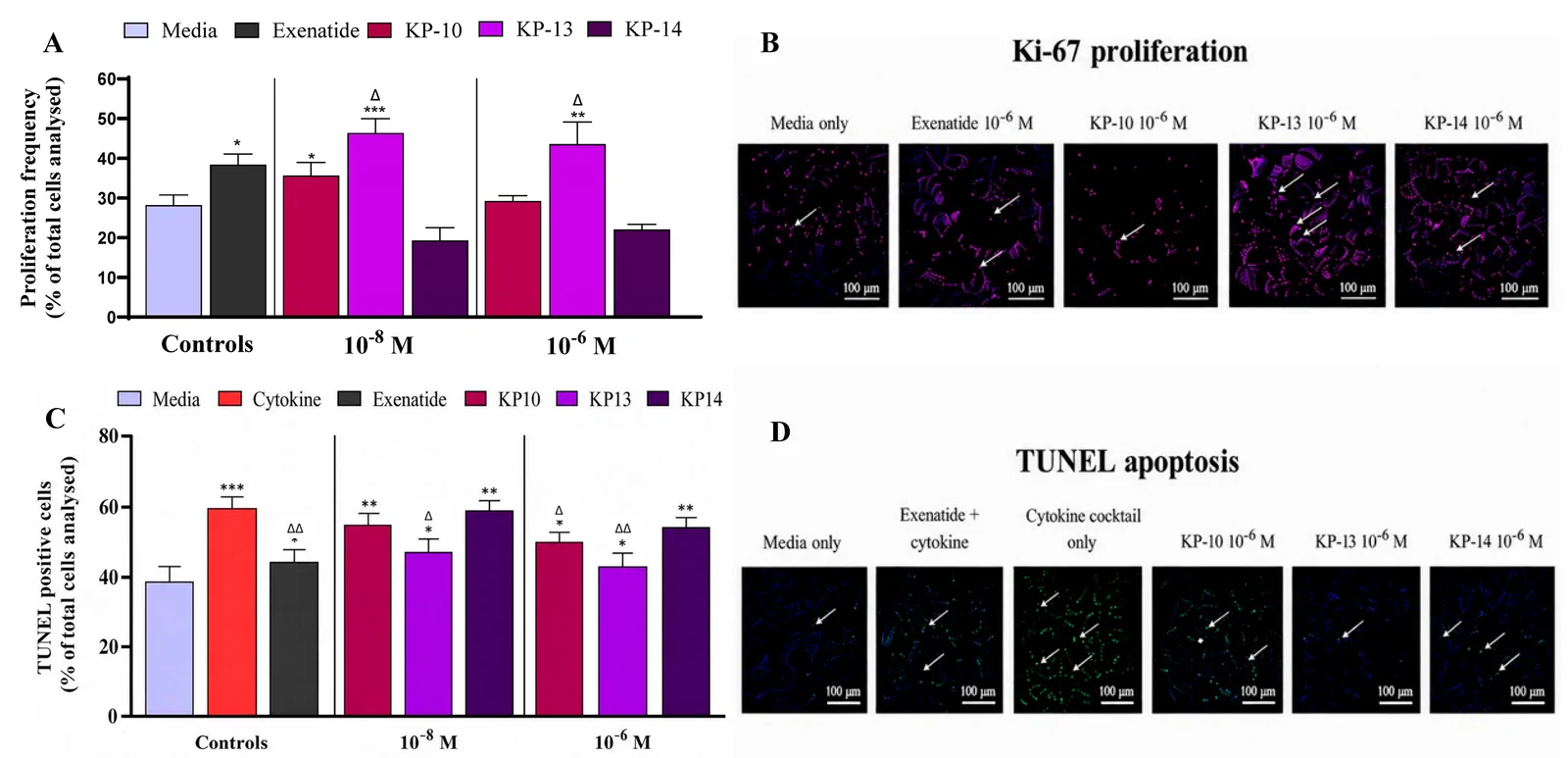
Effects of kisspeptin isoforms on BRIN-BD11 beta-cell proliferation and protection from cytokine-induced apoptosis. (**A**) BRIN-BD11 cells were cultured (18 h) with KP-14, KP-13, KP-10 or exenatide (10^−8^ and 10^−6^ M) and proliferation was assessed by Ki-67 staining. (**C**) BRIN-BD11 cells were cultured (18 h) with KP-14, KP-13, KP-10 or exenatide (10^−8^ and 10^−6^ M) in the presence of a cytokine cocktail (IL-1β (100 U/mL), IFNγ (20 U/mL), TNFα (200 U/mL)) and apoptosis was detected using the TUNEL assay. (**B**,**D**) Representative images of cells positively stained for (**B**) Ki-67 and (**D**) TUNEL from each peptide treatment group, with arrows indicating positively stained cells. All values are mean ± SEM (*n* = 3). * *p* < 0.05, ** *p* < 0.01, *** *p* < 0.001 compared with respective media-alone culture. (**A**) ^Δ^ *p* < 0.05 compared with exenatide and (**C**) ^Δ^ *p* < 0.05, ^ΔΔ^ *p* < 0.01 compared with cytokine cocktail.

Both KP-10 and KP-13 significantly augmented BRIN-BD11 cell proliferation (*p* < 0.01–0.001) at 10^−8^ M (Figure 2A). KP-13 also possessed similar significant beta-cell proliferative benefits (*p* < 0.01) at 10^−6^ M when compared to media-alone control (Figure 2A). Encouragingly, these positive effects of KP-13 on beta-cell growth were more efficacious (*p* < 0.05) than exenatide at both 10^−8^ and 10^−6^ M (Figure 2A). In terms of protection against beta-cell death, as expected, 10^−6^ M exenatide was associated with a partial normalisation of cytokine-induced apoptosis (Figure 2C). All kisspeptin peptides, and especially KP-13, were associated with similar benefits on BRIN-BD11 beta-cell survival (Figure 2C). Representative images of Ki-67- and TUNEL-stained BRIN-BD11 beta-cells from each treatment group are presented in Figure 2B,D, respectively. The arrows indicate positively co-stained insulin and Ki-67 or TUNEL cells, as appropriate.

### 3.5. Kisspeptin Peptides Elevate Blood Glucose Levels and Perturb Glucose Tolerance, but Can Suppress Appetite in Mice

**Figure 3:**
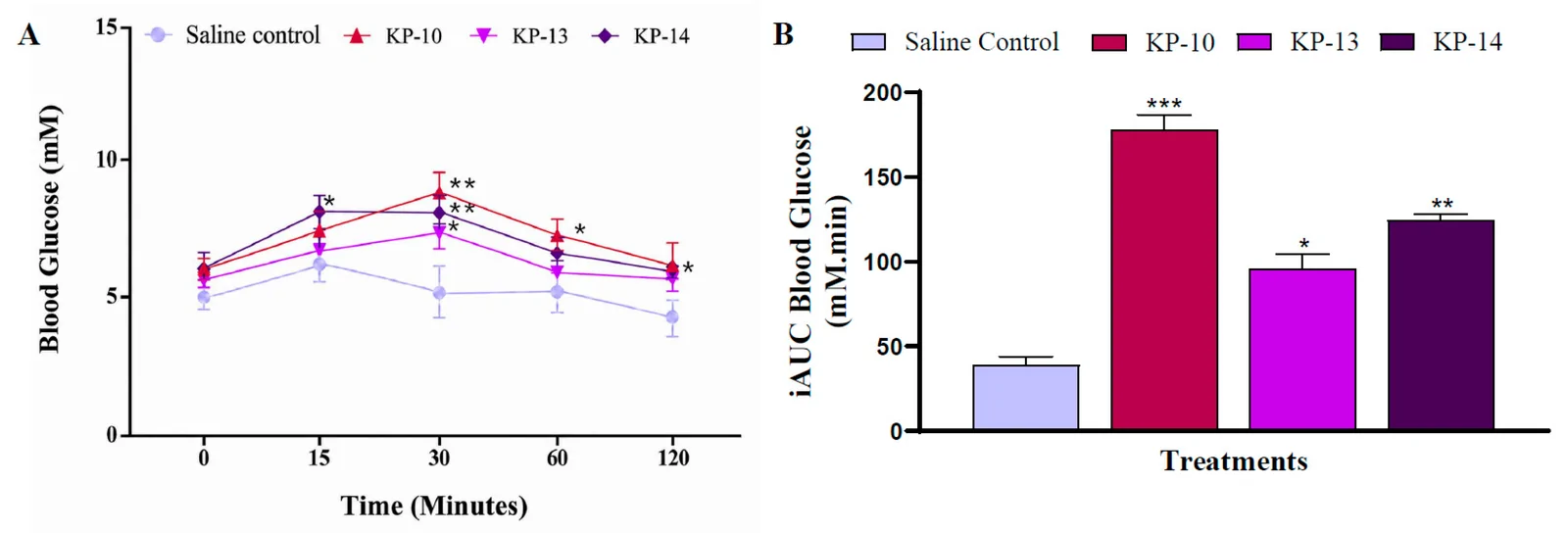
Effects of kisspeptin isoforms on blood glucose levels in male and female mice. (**A**) Blood glucose was assessed immediately prior to, and at regular intervals subsequent to, i.p. injection of KP-14, KP-13 or KP-10 (each at 25 nmol/kg bw) with saline (0.9% NaCl) vehicle. (**B**) Associated 0–120 min AUC data are also presented. All values are mean ± SEM (*n* = 6). * *p* < 0.05, ** *p* < 0.01, *** *p* < 0.001 compared with the saline control group.

**Figure 4:**
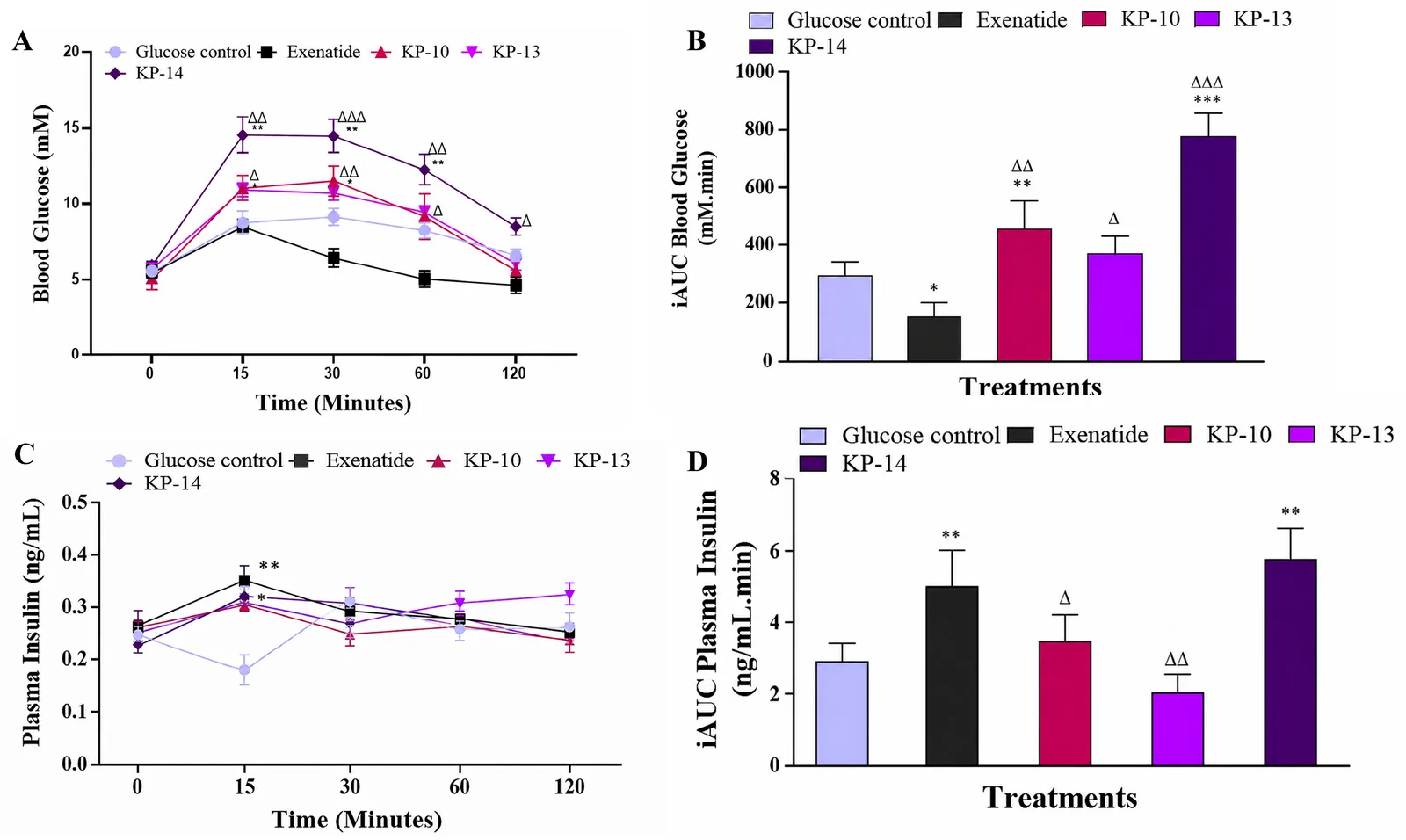
Effects of kisspeptin isoforms on glucose tolerance and glucose-stimulated insulin secretion in male and female mice. (**A**,**C**) Blood glucose and plasma insulin were assessed immediately prior to, and at regular intervals subsequent to, i.p. injection of glucose alone (18 mmol/kg bw) and in combination with KP-14, KP-13, KP-10 or exenatide (each at 25 nmol/kg bw). (**B**,**D**) Associated glucose and insulin 0–120 min AUC data are also presented. All values are mean ± SEM (*n* = 6). * *p* < 0.05, ** *p* < 0.01, *** *p* < 0.001 compared with the glucose control group. ^Δ^ *p* < 0.05, ^ΔΔ^ *p* < 0.01, ^ΔΔΔ^ *p* < 0.001 compared with exenatide.

**Figure 5:**
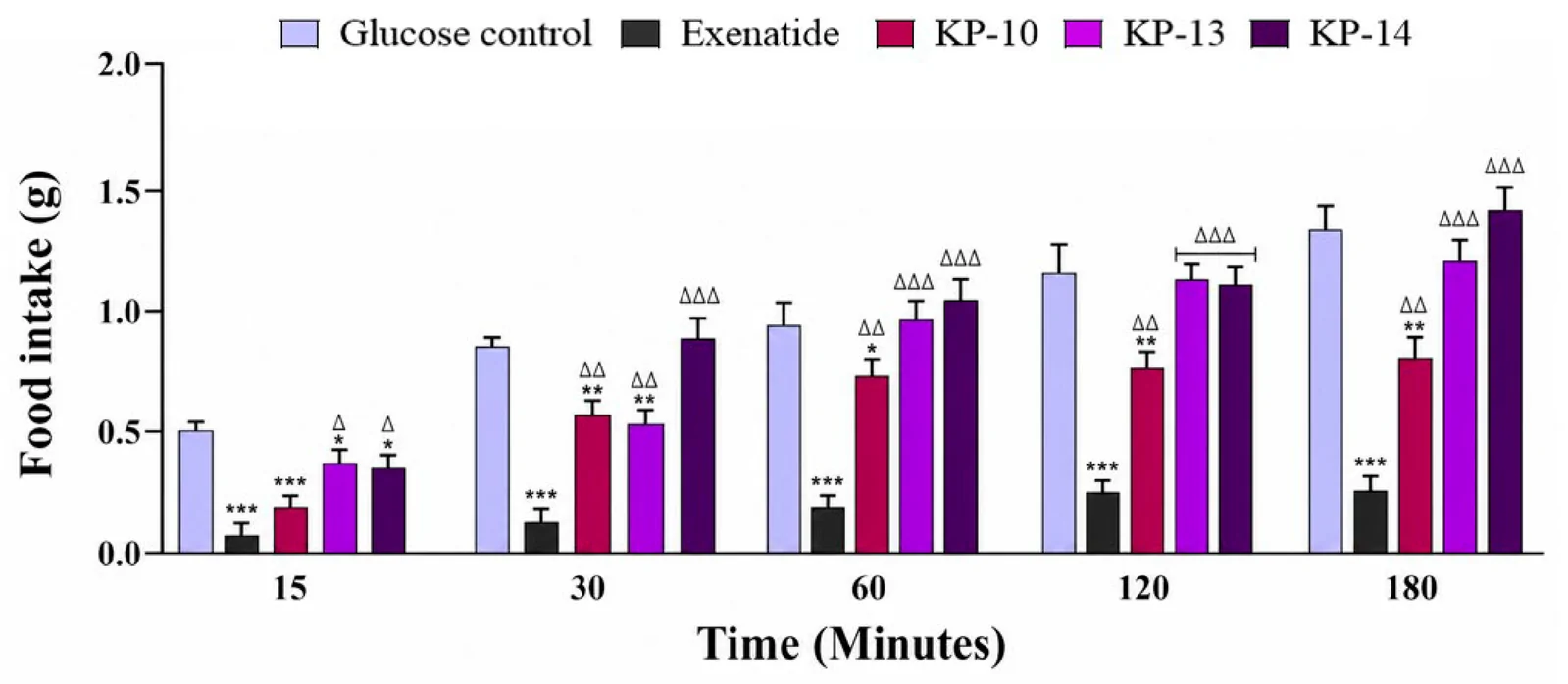
Effects of kisspeptin isoforms on food intake in male and female mice. Cumulative food intake was assessed after i.p. administration of saline vehicle (0.9% NaCl) alone and in combination with KP-14, KP-13, KP-10 or exenatide (each at 25 nmol/kg bw). All values are mean ± SEM (*n* = 6). * *p* < 0.05, ** *p* < 0.01, *** *p* < 0.001 compared with the saline control group. ^Δ^ *p* < 0.05, ^ΔΔ^ *p* < 0.01, ^ΔΔΔ^ *p* < 0.001 compared with exenatide.

Acute administration of 25 nmol/kg KP-14, KP-13 or KP-10 in mice led to small but significant elevations (*p* < 0.05–0.001) of circulating, and especially 0–120 min overall AUC, glucose values when compared to saline vehicle control (Figure 3A,B). As would be expected, following conjoint injection with glucose, exenatide reduced individual blood glucose concentrations, which was associated with a significant decrease (*p* < 0.05) in overall AUC glucose when compared to glucose alone (Figure 4A,B). This effect was accompanied by a clear augmentation (*p* < 0.01) of glucose-induced insulin secretion by exenatide (Figure 4C,D). In contrast, all kisspeptin peptides elevated (*p* < 0.05–0.01) individual blood glucose levels in mice following combined administration with glucose (Figure 4A). However, in terms of 0–120 min AUC values, only KP-10 and KP-14 were associated with increased (*p* < 0.01–0.001) blood glucose levels when compared to glucose-alone control (Figure 4B). Whilst mice treated with KP-13 had similar overall glucose values to the control group, these were still elevated (*p* < 0.05) when compared to exenatide-treated mice (Figure 4B). Interestingly, KP-14 appeared to augment (*p* < 0.01) glucose-induced insulin concentrations, whereas KP-10 and KP-13 had limited effects on insulin secretion (Figure 4D). In relation to food intake, all test peptides, including exenatide, KP-14, KP-13 and KP-10, inhibited (*p* < 0.05–0.001) appetite 15 min post-injection in overnight-fasted mice at a dose of 25 nmol/kg (Figure 5). This appetite-suppressive effect was maintained at 30 min for exenatide, KP-13 and KP-10, but not KP-14 (Figure 5). Indeed, exenatide and KP-10 were associated with distinct reductions (*p* < 0.01–0.001) in food intake across the full 180 min observation period, although KP-10 was less efficacious (*p* < 0.01) than exenatide (Figure 5). KP-13 and KP-14 were devoid of effects on appetite control from 60 min onwards (Figure 5).

## 4. Discussion

As previously documented in related pancreatic beta-cell lines [7,9], we were able to detect KISS1R expression in BRIN-BD11 beta-cells. Notably, KISS1R has also been demonstrated in rodent and human pancreatic islets [7,9], confirming an important physiological role for kisspeptin in islet function and metabolism. Furthermore, fluorescent peptide-binding and cellular imaging studies additionally confirm functional KISS1R proteins on pancreatic islet cells [28], supporting the usefulness of our gene expression studies. There is evidence for kisspeptin co-localisation with insulin and glucagon within islets [7], suggesting that kisspeptin could be considered a non-classical islet peptide, similar to the incretin hormones [29]. Whilst these and related studies help establish kisspeptin as a direct modulator of metabolism, as well as reproductive function [30], limited information is available in terms of whether these biological actions extend across all kisspeptin peptide isoforms.

Kisspeptin peptides have previously been shown to be susceptible to enzymatic proteolysis, with particular emphasis on disruption of the peptide bond between Gly^11^ and Leu^12^ (Table 1) [31]. Yet, in our hands, we were unable to detect Gly^11^-Leu^12^ cleavage in plasma; this may be because our in vitro assay neither fully replicates in vivo degradation processes nor determines systemic peptide half-life. However, as reported by others [32], N-terminal amino acid deletion was observed for KP-13 and KP-10, likely as a result of aminopeptidase action. Of interest, a KP-13 degradation product, namely KP(4-13), possessed an identical amino acid sequence to native KP-10, suggesting that some of the observed bioactivity of KP-13 could be related to formation of KP-10 [6]. However, detectable levels of native KP-13 were apparent even after a 2 h incubation in plasma and, notably, KP-10 generation remains unquantified. Whether other KP-13 and KP-10 plasma degradation products have inherent biological activity remains to be determined, but given that all retain the Phe-Gly-Leu-Arg-Phe-NH_2_ motif known to be critical for KISS1R activation [33], this could be a distinct possibility. Interestingly, KP-14 remained entirely intact after a 2 h incubation in plasma, indicating that the carboxymethyl side-chain group and resulting negative charge of Asp can afford some protection against aminopeptidase. In some agreement, the biological half-life of KP-54 was noted to be significantly protracted when compared to KP-10 [34], consistent with the idea that larger kisspeptin peptides are more stable. However, related studies with KP-54 in the current setting would be required to substantiate this suggestion. Furthermore, characterising the in vivo pharmacokinetic profile and tissue distribution of these kisspeptin isoforms would help complement interpretation of our enzymatic stability data, but such studies fall outside the scope of the current study.

There are conflicting views on the effects of kisspeptin peptides on insulin secretion, with inhibitory effects reported at nanomolar concentrations [10,35] but stimulation at micromolar peptide concentrations [7,36]. In some agreement, KP-14 inhibited insulin release from BRIN-BD11 cells at 10^−9^ and 10^−8^ M, with similar effects of KP-10 at 10^−10^ and 10^−7^ M. In contrast, there was a small, but significant, augmentation of beta-cell secretory function by KP-13 at 10^−10^ M, adding to the idea that the impact of kisspeptin peptides on beta-cell function is complex. In this regard, paracrine signalling has been suggested to play an important role in the regulation of insulin secretion by kisspeptin [37]. Thus, in agreement with our observations in isolated murine islets, beta-cell stimulatory effects of kisspeptin peptides may be dependent on paracrine signalling within the intact islet microenvironment [38], which is absent in BRIN-BD11 cells. Augmented beta-cell secretory capacity would certainly be considered as an advantage in diabetes-related diseases [39]. That said, acute stimulation of insulin secretion is unlikely to represent the sole biological action of kisspeptin on pancreatic beta-cells and islets.

In this regard, an important unmet need in type 2 diabetes pharmacotherapy is the long-term preservation and restoration of functional beta-cell mass [39]. Whilst incretin therapies were once considered to hypothetically address this issue [40], translation to the human setting is largely missing [41]. Remarkably, KP-13 produced greater Ki-67 positivity than the same concentration of exenatide tested under our in vitro conditions in BRIN-BD11 cells. When this is coupled with protective effects against cytokine-induced beta-cell apoptosis equivalent to exenatide, KP-13 demonstrates potential capacity to exert beta-cell health benefits. Such effects are most plausibly mediated through KISS1R-coupled activation of PI3K/Akt pathways, which suppresses expression of pro-apoptotic Bcl-2 family members, including BAD and BAX [42]. Furthermore, concurrent KISS1R activation of the MAPK/ERK cascade has been proposed to reinforce downstream cell-survival mechanisms [43]. However, these mechanistic observations were derived from non-islet cell types. Since the current study did not directly measure pathway activation within pancreatic islets, these suggested mechanisms remain strictly hypothetical. Assessment of the secretory function of any newly divided beta-cells, or those protected from apoptosis, would be required to fully ascertain the physiological importance of KP-13-mediated effects on beta-cell turnover. Whilst KP-10 and KP-14 also imparted some beta-cell survival actions, proliferative effects were less apparent, apart from KP-10 at 10^−8^ M. The reason for such discrepancies in bioactivity of the kisspeptin peptide isoforms is not altogether obvious but may be related to three-dimensional structures that affect receptor binding affinity and activation of specific second messenger pathways.

Reminiscent of observations in BRIN-BD11 cells, in vivo investigations supported the notion that the kisspeptin isoforms exert differential effects on insulin secretion. To account for potential sex differences [44], and in line with current standards for improving the translational potential of animal experimentation [45,46], we employed equal numbers of male and female mice for all studies. In good agreement with others [8], KP-14 significantly enhanced glucose-induced insulin secretion. However, KP-10 and KP-13 were devoid of beta-cell stimulatory actions in this setting. There is evidence to indicate that kisspeptin enhances insulin action [8], which may be important for overall effects on glucose homeostasis, especially since KP-13 demonstrated insulinotropic actions in isolated pancreatic islets. However, we are unable to perform euglycemic–hyperinsulinaemic clamp experiments, and our rodent model had intact insulin signalling, meaning further studies are required to fully investigate this facet of kisspeptin bioactivity. The contrasting effects of KP-13 on insulin secretion from BRIN-BD11 cells, isolated islets and mice are intriguing and may be related to aspects such as rapid peptide clearance or the presence of intact neural and vascular regulatory networks in the living organism. Nonetheless, all kisspeptin peptides tended to elevate overall AUC blood glucose levels in mice, whether injected conjointly with saline or glucose. The only exception was KP-13, which correlates with more distinct effects of this kisspeptin isoform in BRIN-BD11 cells, and positions this kisspeptin isoform as a promising candidate for potential therapy for type 2 diabetes.

Interestingly, in overnight-fasted mice all kisspeptin isoforms suppressed appetite 15 min post-injection, in line with earlier studies in rodents [14]. However, clinical trials in both men and women would suggest that kisspeptin does not significantly affect appetite [13,47], consistent with the effects of KP-14 and KP-13 at later timepoints in our experimental system. Although somewhat speculative, our findings might suggest that KP-10 is the primary driver of appetite suppression of kisspeptin, perhaps through enhanced brain penetration of the smaller peptide isoform [48]. Indeed, enzymatic cleavage of KP-13, but not KP-14, from KP-10 would concur with this notion. Of note, all in vivo studies were performed in healthy mice and therefore do not establish whether such actions translate to the obesity or type 2 diabetes settings, where alterations in beta-cell mass, insulin sensitivity and endogenous kisspeptin signalling may modify peptide isoform actions. In addition, our relatively small sample size for in vivo experiments and the crossover design could also be regarded as limitations in terms of translation. However, our findings certainly have clinically relevant implications, considering that pancreatic beta-cell GPR54 protein expression is downregulated in obese and type 2 diabetic rodents [49], and that GPR54 knockout can lead to obesity and glucose intolerance [50]. In addition, circulating kisspeptin levels are decreased in women with obesity and polycystic ovary syndrome [51]. Together, these findings point toward the potential benefits of kisspeptin-based therapies for metabolic disease.

## 5. Conclusions

In conclusion, the present study demonstrates that kisspeptin peptide isoforms possess distinct metabolic profiles. Although KP-14 displayed complete resistance to plasma degradation in our in vitro system and enhanced glucose-stimulated insulin secretion in vivo, this was accompanied by impaired glucose homeostasis and only modest beneficial effects on beta-cell health. In contrast, KP-13 consistently augmented beta-cell proliferation and protected against cytokine-induced apoptosis, promoted insulin secretion from isolated islets and did not perturb glucose tolerance, whilst also encouraging appetite suppression. Interestingly, the bioactivity profile of KP-13 could be partly linked to enzymatic generation of KP-10 in plasma, but this link requires further validation since KP-10 generation has not yet been quantified. Moreover, the aforementioned appetite-suppressive effects of KP-13 were short-lived, and its in vivo insulinotropic actions were absent in mice. Collectively, our findings demonstrate that the metabolic actions of kisspeptin cannot be generalised across peptide isoforms. Overall, the current study helps facilitate the rational development of kisspeptin-based therapeutics for obesity-driven forms of diabetes such as type 2 diabetes, to maximise beneficial metabolic actions while minimising undesirable effects on glucose homeostasis.

## Figures and Tables

**Table 1:** Overview of murine plasma degradation profile for KP-14, KP-13 and KP-10.

	Peptide Detected at 0 h	Peptide Detected at 2 h	Amino Acid Sequence	Retention Time (min)	Calculated Mass (Da)	Theoretical Mass (Da)
Kisspeptin-14	KP-14		D-L-P-N-Y-N-W-N-S-F-G-L-R-F	24.77	1741.91	1741.90
		KP-14	D-L-P-N-Y-N-W-N-S-F-G-L-R-F	24.76	1741.93	1741.90
Kisspeptin-13	KP-13		L-P-N-Y-N-W-N-S-F-G-L-R-F	24.76	1627.19	1626.85
		KP-13	L-P-N-Y-N-W-N-S-F-G-L-R-F	24.77	1626.83	1626.85
		KP(3-13)	N-Y-N-W-N-S-F-G-L-R-F	24.13	1416.71	1416.90
		KP(4-13)	Y-N-W-N-S-F-G-L-R-F	23.92	1302.65	1302.45
		KP(5-13)	N-W-N-S-F-G-L-R-F	23.41	1139.63	1139.20
Kisspeptin-10	KP-10		Y-N-W-N-S-F-G-L-R-F	24.04	1302.66	1302.45
		KP(2-10)	N-W-N-S-F-G-L-R-F	24.13	1139.64	1139.27
		KP(3-10)	W-N-S-F-G-L-R-F	23.39	1025.27	1025.27

## Data Availability

The authors declare that the data supporting the findings of this study are available within the article. Any additional raw data supporting the conclusions of this article will be made available by the lead author, without undue reservation.

## Supplementary Materials

The supporting information can be downloaded at https://www.mdpi.com/article/10.3390/metabo16090686/s1. Supplementary Figure S1. Representative HPLC traces for murine plasma degradation of KP-14 at 0 and 2 h. Supplementary Figure S2. Representative HPLC traces for murine plasma degradation of KP-13 at 0 and 2 h. Supplementary Figure S3. Representative HPLC traces for murine plasma degradation of KP-10 at 0 and 2 h.
