# Supplementary material for: Enzymatic Stability and Comparative Effects of Kisspeptin-14, Kisspeptin-13 and Kisspeptin-10 on Beta-Cell Function, Glucose Homeostasis and Appetite Regulation
Source: Metabolites. 2026 Sep 17;16(9):686. doi: 10.3390/metabo16090686 (PMC13609550; doi:10.3390/metabo16090686)
Supplement: Supplementary file 1 [file metabolites-16-00686-s001.zip › metabolites-4542271-supplementary.pdf]

Supplementary Figure S1. Representative HPLC traces for murine plasma degradation of KP-14 at (A) 0 and (B) 2 hours.

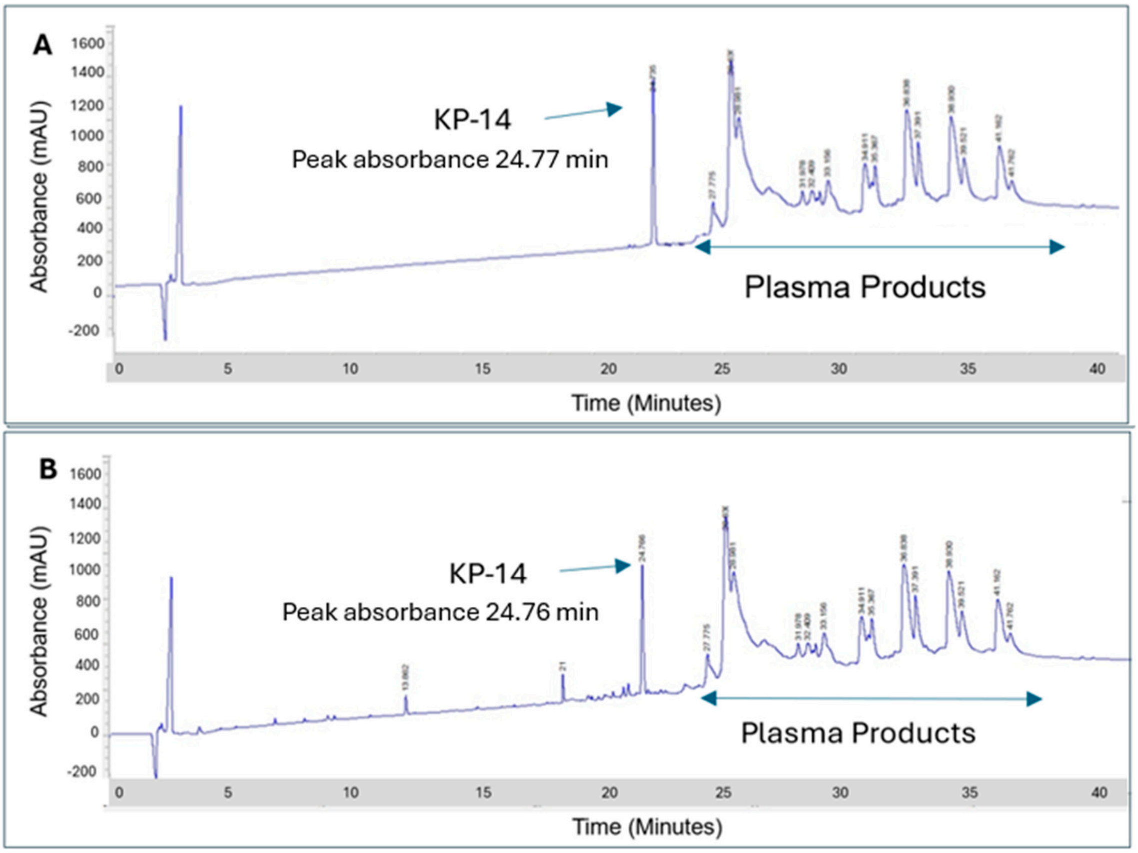

Peptides (50 μg) were incubated with 10 μl fasted murine plasma for 0 and 2 hours. Reactions were terminated by addition of trifluoroacetic acid (10% v/v). Degradation profiles were analysed using rp-HPLC. Elution gradient of solvent B (350 ml acetonitrile, 150 ml di$H_2$0, 500 μl Trifluoracetic acid) was 0% at 0 min, 0% - 44% over 10 minutes, 40% - 70% over 40 minutes and 70% - 100% over 10 minutes. Absorbance was monitored at 214 nm using Agilent 1100 Series Variable Wavelength Detector (VWD).

Supplementary Figure S2. Representative HPLC traces for murine plasma degradation of KP-13 at (A) 0 and (B) 2 hours.

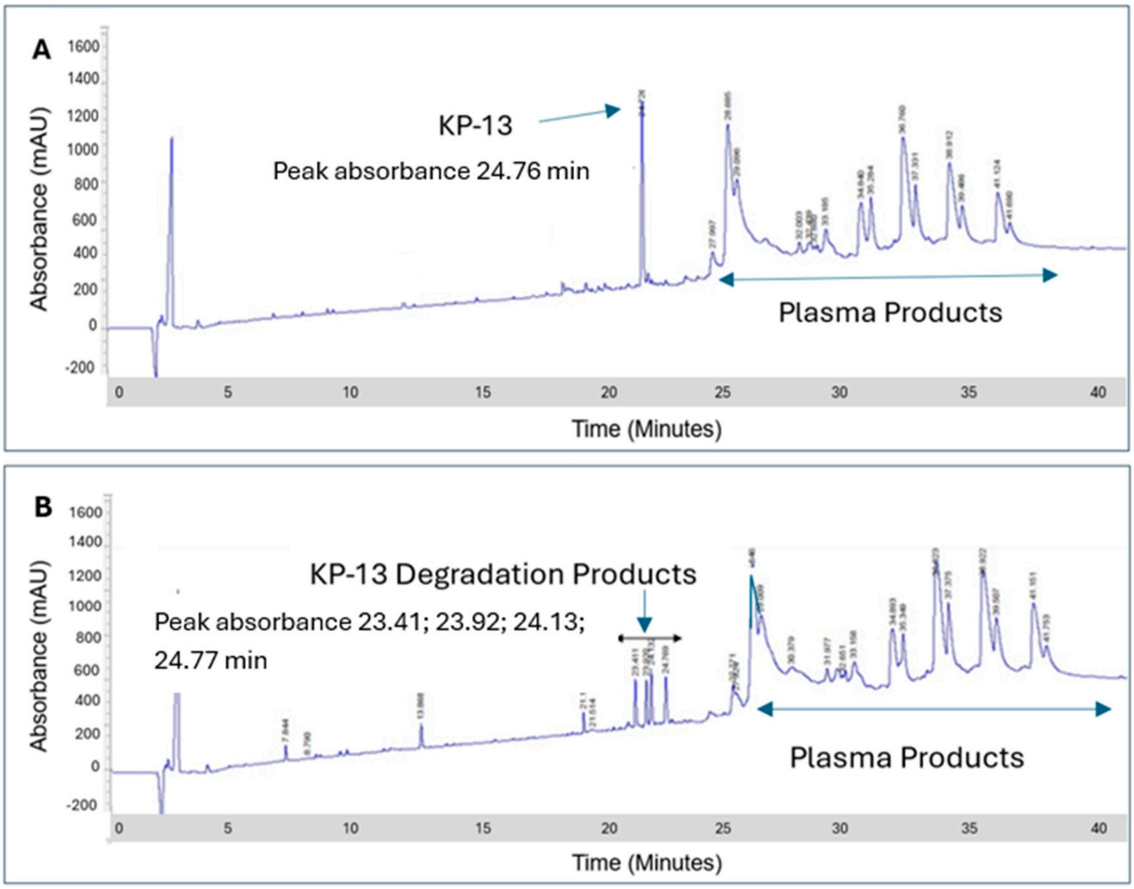

Peptides (50 µg) were incubated with 10 µl fasted murine plasma for 0 and 2 hours. Reactions were terminated by addition of trifluoroacetic acid (10% v/v). Degradation profiles were analysed using rp-HPLC. Elution gradient of solvent B (350 ml acetonitrile, 150 ml diH_20, 500 µl Trifluoracetic acid) was 0% at 0 min, 0% - 44% over 10 minutes, 40% - 70% over 40 minutes and 70% - 100% over 10 minutes. Absorbance was monitored at 214 nm using Agilent 1100 Series Variable Wavelength Detector (VWD).

Supplementary Figure S3. Representative HPLC traces for murine plasma degradation of KP-10 at (A) 0 and (B) 2 hours.

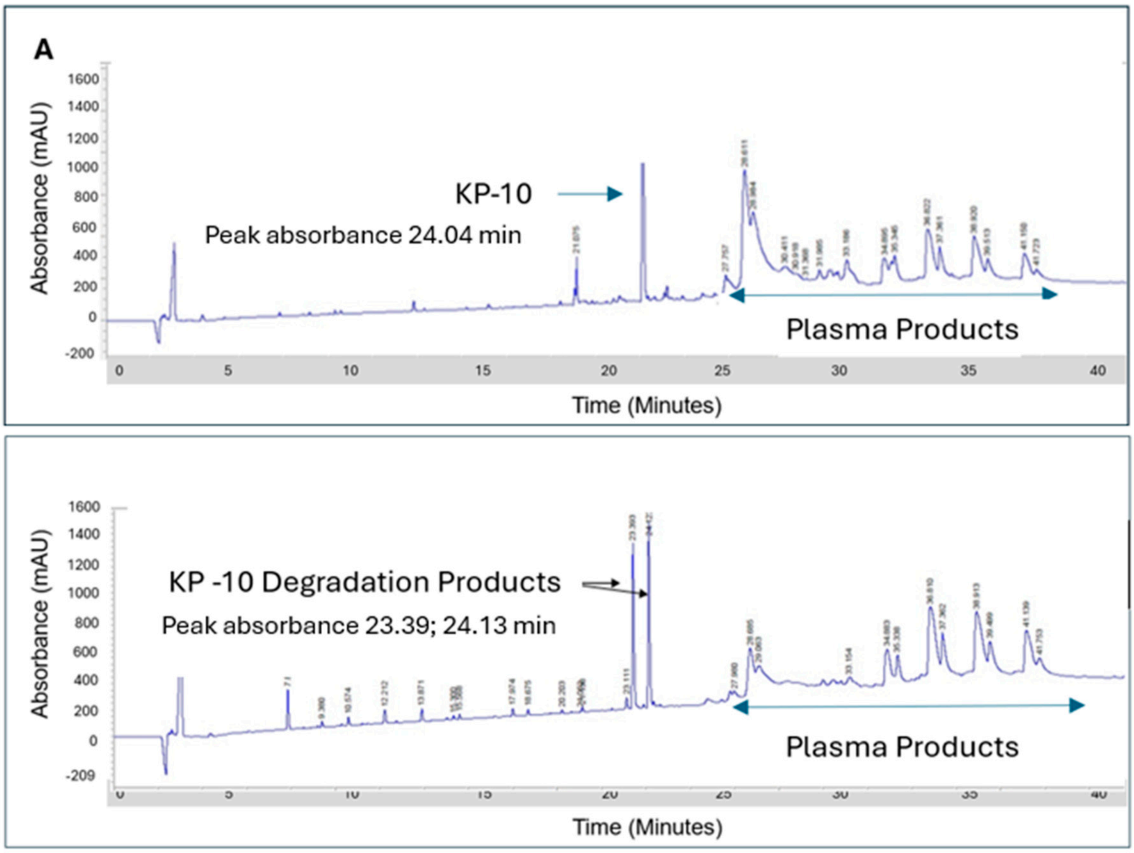

Peptides (50 μg) were incubated with 10 μl fasted murine plasma for 0 and 2 hours. Reactions were terminated by addition of trifluoroacetic acid (10% v/v). Degradation profiles were analysed using rp-HPLC. Elution gradient of solvent B (350 ml acetonitrile, 150 ml diH$_2$0, 500 μl Trifluoracetic acid) was 0% at 0 min, 0% - 44% over 10 minutes, 40% - 70% over 40 minutes and 70% - 100% over 10 minutes. Absorbance was monitored at 214 nm using Agilent 1100 Series Variable Wavelength Detector (VWD).
